# Impacts of COVID-19 on mothers’ and newborns’ health outcomes in regional Canada: A cross-sectional analysis

**DOI:** 10.1016/j.heliyon.2024.e34165

**Published:** 2024-07-05

**Authors:** Stefan Kurbatfinski, Aliyah Dosani, Carlos Fajardo, Alexander Cuncannon, Aliza Kassam, Abhay K. Lodha

**Affiliations:** a Owerko Centre, Alberta Children’s Hospital Research Institute, University of Calgary, 2500 University Drive NW, Calgary, AB, T2N 1N4, Canada; b Department of Community Health Sciences, Cumming School of Medicine, University of Calgary, 3330 Hospital Dr NW, Calgary, AB, T2N 4N1, Canada; c Faculty of Health, Community and Education, Mount Royal University, 4825 Mount Royal Gate SW, Calgary, AB, T3E 6K6, Canada; d O'Brien Institute for Public Health, University of Calgary, 3280 Hospital Dr NW, Calgary, AB, T2N 4Z6, Canada; e Department of Pediatrics, Cumming School of Medicine, University of Calgary Foothills Medical Centre, 1403 29 St NW, Calgary, AB, T2N 2T9, Canada; f Addiction and Mental Health, Alberta Health Services, Calgary, AB, Canada; g Department of Medical Sciences, Western University, 1151 Richmond St, London, ON, N6A 3K7, Canada; h Department of Pediatrics and Community Health Sciences, Alberta Children's Hospital Research Institute, Cumming School of Medicine, University of Calgary, 3330 Hospital Dr NW, Calgary, AB, T2N 4N1, Canada

**Keywords:** COVID-19, Hospital use, Pregnancy outcomes, Newborn health, Maternal health

## Abstract

**Background:**

COVID-19 infection and pandemic-related stressors (e.g., socioeconomic challenges, isolation) resulted in significant concerns for the health of mothers and their newborns during the perinatal period. Therefore, the primary objective of this study was to compare the health outcomes of pregnant mothers and their newborns one year prior to and one year into the pandemic period in Alberta, Canada. Secondary objectives included investigating: 1) predictors of admission to neonatal intensive care units (NICU) and to compare NICU-admitted newborn health outcomes between the two time periods; 2) hospital utilization between the two time periods; and 3) the health outcomes of mothers and their newborns following infection with COVID-19.

**Methods:**

This analytical cross-sectional study used a large administrative dataset (n = 32,107) obtained from provincial regional hospitals and homebirths in Alberta, Canada, from April 15, 2019, to April 14, 2021. Descriptive statistics characterized the samples. Chi-squares and two-sample t-tests statistically compared samples. Multivariable logistic regression identified predictor variables.

**Results:**

General characteristics, pregnancy and labor complications, and infant outcomes were similar for the two time periods. Preterm birth and low birthweight predicted NICU admission. During the pandemic, prevalence of hospital visits and rehospitalization after discharge decreased for all infants and hospital visits after discharge decreased for NICU-admitted neonates. The odds of hospital revisits and rehospitalization after discharge were higher among newborns with COVID-19 at birth.

**Conclusions:**

Most of the findings are contextualized on pandemic-related stressors (rather than COVID-19 infection) and are briefly compared with other countries. Hospitals in Alberta appeared to adapt well to COVID-19 since health conditions were comparable between the two time periods and COVID-19 infection among mothers or newborns resulted in few observable impacts. Further investigation is required to determine causal reasons for changes in hospital utilization during the pandemic and greater birthweight among pandemic-born infants.

## 1 Introduction

When the World Health Organization (WHO) declared the global pandemic of coronavirus disease 2019 (COVID-19) in 2020 [1], nearly all sectors of society were forced to quickly adapt [2]. In addition to healthcare changes and lockdown measures, financial and employment loss or change, transitions to online schooling, and work-from-home orders exacerbated the number of stressors that individuals experienced, leading to an observed increase in adverse mental (e.g., depression) and physical (e.g., weight change) health outcomes globally [2]. The impacts of pandemic restrictions and the associated stress on pregnant mothers and newborn infants are particularly important to investigate given positive associations between prenatal stress and increased risk of negative health consequences for both mothers and their children across the lifespan [3]. Further, fears around contraction of COVID-19 within healthcare settings and changes in healthcare policy also suggest implications on hospital utilization which can undermine the acquisition of adequate healthcare [4]. In addition to the impacts deriving from prenatal stress, COVID-19 infection among pregnant women or their newborns has been associated with maternal and newborn health problems [5], revealing a direct and important mechanism through which the pandemic can impact mother-newborn health and increase the need for hospital use (e.g., admission to neonatal intensive care units (NICUs)) [6]. Collectively, the changes arising from the COVID-19 pandemic challenged how women navigated their pregnancy, suggesting potential health repercussions among mothers and their newborns that require further investigation.

It is well understood that mothers' prenatal stress is positively associated with adverse health outcomes among their children, including, but not limited to, cognitive deficiencies, respiratory problems, and a weakened immune system [7,8]. The effects of prenatal stress are characterized by their effects on fetal and infant neurodevelopment and physiological functioning, through which prenatal stress undermines typical development of brain structure and function [9]. For example, female children born to mothers who experienced prenatal stress exhibit larger right amygdala lobes which in turn is associated with greater stress response activation [10]. Other health problems in children, such as respiratory problems, also arise when exposed to stressful conditions during their mother's pregnancy [11]. Since the emergence of the COVID-19 pandemic resulted in numerous stressors that underlie family dysfunction (e.g., financial instability) [12], pregnant mothers were more likely to experience prenatal stress [13], suggesting an indirect mechanism through which the pandemic may have impacted the health of mothers and their newborns. In fact, a population-based study conducted in Canada reported that nearly 50 % and 35 % of pregnant women experienced clinically relevant symptoms of anxiety and depression, respectively, during the COVID-19 pandemic [14], revealing the importance of examining the health of mothers and their newborns during the pandemic.

In addition, turbulent policies and regulations related to COVID-19 alongside fears of contracting COVID-19 likely discouraged pregnant women from accessing healthcare settings due to confusion [15]. In Alberta, patients visiting hospitals were only allowed one visitor for an hour at allocated times, which decreased patients’ perceived level of social support. Additionally, lockdown restrictions were introduced and removed several times, likely causing confusion among Albertan residents regarding healthcare access. These factors point to the need to examine hospital utilization during the pandemic to shed light on how healthcare was used during this critical time period and to hypothesize implications on the health of mothers and their newborns.

Alongside indirect effects of the pandemic on the health of mothers and their newborns, contraction of COVID-19 by mothers who are pregnant has been directly associated with several adverse outcomes for both mothers and infants [16]. For example, infection with COVID-19 among pregnant women has been linked to more preterm births and cesarean deliveries [17,18]; mothers also required supplemental resources (e.g., oxygen supplies) due to COVID-19-related symptoms [9]. Further, pregnant women who contracted COVID-19 were observed to have increased adverse health conditions compared to their non-infected counterparts, including hypertensive disorders and maternal death [5,19]. A nationally representative prospective cohort conducted in England also showed how newborns born to mothers with COVID were at-risk of neonatal adverse outcomes (e.g., very low birthweight, respiratory distress syndrome, seizures) than those born to non-infected mothers [5]. There is a smaller body of evidence revealing vertical transmission of COVID-19 from mothers to their newborns; however, in cases where newborns did contract COVID-19 and also exhibited severe symptoms, neonatal death was more likely to occur [20]. Given that considerable evidence reveals how contraction of the COVID-19 virus negatively impacts the health of both mothers and newborns, the capacity for healthcare systems to quickly adapt and provide adequate care is essential to help buffer these negative impacts.

Studies that have examined the effects of the COVID-19 pandemic on pregnancy, mother, and newborn outcomes reveal differences based on country and regions. For example, Dandona et al. observed a significant increase in the incidence of adverse pregnancy outcomes (e.g., stillbirths, neonatal deaths) in their Indian-based study (a low-to-middle income country) [21], while Liu et al. observed few increases in health conditions with the onset of the pandemic in their Canadian-based study (a high-income country) [22]. These patterns are reiterated through a meta-analysis conducted by Gajbhiye et al. [23], whereby the overall risk of adverse pregnancy outcomes was higher among low-to-middle income countries than in high-income countries. It is important to note that within country differences are also observed which can be attributed to regional differences. For example, impoverished, marginalized, and/or rural communities are less likely to ascertain adequate acquisition of healthcare services and resources, leading to regional health inequities [24]. Therefore, it is important to consider socioeconomic contexts when interpreting the impacts of COVID-19 on mothers and their newborns.

As emerging data demonstrate regional differences regarding the effects of COVID-19 on various pregnancy outcomes [23], data from different countries and regions are needed to best understand the impacts of the pandemic on pregnant women and their newborns across the globe. This manuscript adds to existing COVID-19 pregnancy literature by using an impressively large dataset to enrich understandings of the impacts of COVID-19 on mother-newborn health in regional Alberta, Canada and to facilitate cross-regional comparisons. The primary objective of this study was to compare the health outcomes of mothers and their newborns one year prior to and one year into the pandemic period in Alberta, Canada. Secondary objectives included investigating: 1) predictors of admission to NICU and to compare NICU-admitted newborn health outcomes between the two time periods; 2) hospital use patterns between the two time periods; and 3) the health outcomes of mothers and their newborns following infection with COVID-19. We hypothesized that women who conceived during the pandemic period and delivered during or after pandemic would have more adverse maternal and neonatal outcomes. Regarding secondary objectives, we expected to see more NICU admissions, more health problems among NICU-admitted newborns, and lower levels of hospital use during the pandemic, while also observing worsened health outcomes for mothers and their newborns following infection with COVID-19.

## 2 Methods

### 2.1 Study site and data collection

This study employed a cross-sectional study design, using a large administrative dataset from regional hospitals and home births in Alberta, Canada, collected from April 15, 2019, to April 24, 2021. The study population constituted data from April 15, 2019, to April 14, 2020 (one year prior to the pandemic) and April 15, 2020, to April 14, 2021 (one year into the pandemic). Alberta's primary healthcare is regulated provincially and provided universally to the public for any necessary (e.g., hospital use) and non-specialist (e.g., primary care) appointments at no cost.

Data included information pertaining to mothers' and newborns' outcomes, including late preterm (34^+0^ to 36^+6^ weeks’ gestation) and term (equal to or more than 37 weeks gestation) infants. Variables included gestational age and low birthweight (<2500 g), number of livebirths and stillbirths, mode of delivery (i.e., caesarean, vaginal), NICU admission, other maternal medical conditions (i.e., diabetes and hypertensive disorders including pre-existing hypertension, gestational hypertension, preeclampsia or HELLP syndrome, eclampsia), other newborn morbidities (i.e., hypoglycemia, jaundice/hyperbilirubinemia, transient tachypnoea of newborn, respiratory distress syndrome, meconium aspiration syndrome, sepsis, necrotizing enterocolitis, hypoxic-ischemic encephalopathy, hypothermia), and hospital utilization (i.e., readmission, rehospitalization). Administrative data were collected from hospital patient charts. Medical conditions were identified using classifications based on the International Statistical Classification of Diseased (ICD) and Related Health Problems, 10th Revision, put forth by the WHO, a universal classification system of medical conditions [25].

### 2.2 Data analysis

Statistical analyses were performed using Wizard 2. Descriptive statistics characterized the sample by comparing maternal and infant sociodemographic characteristics. To compare the prevalence of various outcomes prior to and during the COVID-19 pandemic, Chi-square tests were used for categorical data and two-sample t-tests for continuous data. Additionally, to identify predictor variables on outcomes of hospital revisits and rehospitalizations, multivariable logistic regression analyses were employed, and resulting odds ratios (ORs) described the direction (protective versus risk factor) and magnitude of the association. A significance level value of α = 0.05 and 95 % confidence intervals were used for all analyses.

Logistic regression models for all births were adjusted for general characteristics (birth during pandemic, cesarean delivery, and multiple delivery), pregnancy and labour complications (gestational diabetes mellitus, hypertensive disorders of pregnancy, intrauterine growth restriction, and premature rupture of membranes), and infant outcomes (NICU visit, low birthweight, gestational age less than 37 weeks, respiratory distress, meconium aspiration, hyperbilirubinemia, pneumonia, hypoglycemia, hypothermia, hypoxic-ischemic encephalopathy, sepsis, and necrotizing enterocolitis).

## Ethics approval

Data for this study come from Alberta Health Services, following guidelines and privacy policies set forth by the provincial healthcare system to ensure confidentiality and anonymity. As this study used an administrative dataset, no verbal or written consent was obtained. Ethics approval was obtained from the Conjoint Ethics Boards of University of Calgary (REB20-0808_REN2) and Mount Royal University (#102283).

## 3 Results

Of 32,900 initial records, 793 were excluded because of missing data. These reasons included no recorded birth weight (n = 758), discrepancies in recorded birth weight (n = 28), and no recorded gestational age (n = 7). The final analytical sample size consisted of 32,107 newborns.

### 3.1 Primary outcome: mother and newborn outcomes from all births

Maternal and neonatal characteristics were generally comparable for all births (n = 32,107) before and during the COVID-19 pandemic, aside from hospital visits after discharge home within 90 days and rehospitalization after discharge (Table 1). Among all infants, the prevalence of hospital revisits (18.8 % versus 22.2 %; prevalence odds ratio (POR) = 0.81, 95 % CI, 0.77–0.86) and rehospitalization (6.1 % versus 7.5 %; POR = 0.81, 95 % CI, 0.74–0.88) within 90 days decreased for infants born during the COVID-19 pandemic (Table 1). Prevalence of hypertensive disorders of pregnancy (10.4 % versus 9.6 %; POR = 1.09, 95 % CI, 1.02–1.18) and newborns’ respiratory distress (4.9 % versus 4.1 %; POR = 1.21, 95 % CI, 1.09–1.35) slightly increased during the pandemic (Table 1). The proportion of NICU admissions remained comparable (10.4 % versus 10.1 %; Table 1).

**Table 1:** Characteristics and outcomes for all infants (n = 32107).

	No./total (%)				
	Birth during pandemic	Birth before pandemic	Absolute difference (95 % CI)	Prevalence ratio	Prevalence odds ratio (95 % CI)
**General characteristics**					
Cesarean delivery	4680/15574 (30.1)	4893/16533 (29.6)	0.45 (−0.55 to 1.46)	1.02	1.02 (0.97–1.07)
Multiple delivery	422/15574 (2.7)	435/16533 (2.6)	0.08 (−0.27 to 0.43)	1.03	1.03 (0.90–1.18)
**Pregnancy and labor complications**					
GDM	1779/15574 (11.4)	1947/16533 (11.8)	−0.35 (−1.05 to 0.35)	0.97	0.97 (0.90–1.03)
HDP	1623/15574 (10.4)	1591/16533 (9.6)	0.80 (0.14 to 1.46)	**1.08**	**1.09 (1.02–1.18)**
IUGR	1531/15574 (9.8)	1643/16533 (9.9)	−0.11 (−0.76 to 0.55)	0.99	0.99 (0.92–1.06)
PROM	3102/15574 (19.9)	3362/16533 (20.3)	−0.42 (−1.29 to 0.46)	0.98	0.97 (0.92–1.03)
**Infant outcomes**					
NICU visit	1612/15574 (10.4)	1675/16533 (10.1)	0.22 (−0.44 to 0.88)	1.02	1.02 (0.95–1.10)
GA <37 weeks	1119/15574 (7.2)	1145/16533 (6.9)	0.26 (−0.30 to 0.82)	1.04	1.04 (0.96–1.13)
Low birthweight	891/15574 (5.7)	1001/16533 (6.1)	−0.33 (−0.85 to 0.18)	0.94	0.94 (0.86–1.03)
Respiratory distress	762/15574 (4.9)	672/16533 (4.1)	0.83 (0.37 to 1.28)	**1.20**	**1.21 (1.09–1.35)**
**Health care seeking and utilization**					
Visit after discharge home within 90 days	2928/15574 (18.8)	3668/16533 (22.2)	−3.39 (−4.27 to −2.50)	**0.85**	**0.81 (0.77–0.86)**
Rehospitalization after discharge	955/15574 (6.1)	1236/16533 (7.5)	−1.34 (−1.89 to −0.79)	**0.82**	**0.81 (0.74–0.88)**

NOTE: GA = gestational age; GDM = gestational diabetes; HDP = hypertensive disorder of pregnancy; HIE = hypoxic ischemia encephalopathy; IUGR = intrauterine growth restriction; PROM = premature rupture of membranes.

### 3.2 Predictors of NICU admission and outcomes for mothers and their newborn

Infants who had a NICU visit were more likely to be born by cesarean (POR = 1.55, 95 % CI, 1.44–1.67) or multiple (POR = 6.22, 95 % CI, 5.39–7.17) delivery, and their mothers were more likely to experience hypertensive disorders of pregnancy (POR = 2.58, 95 % CI, 2.35–2.84), intrauterine growth restriction (POR = 2.07, 95 % CI, 1.88–2.29), and premature rupture of membranes (POR = 1.30, 95 % CI, 1.19–1.41; Table 2). These infants were also more likely to be born preterm (POR = 23.83, 95 % CI, 21.63–26.25) and have low birthweight (POR = 17.54, 95 % CI, 15.86–19.41), in addition to experiencing greater prevalence of all adverse birth outcomes (Table 2). Lastly, these infants were more likely to have hospital visits (POR = 1.15, 95 % CI, 1.06–1.25) and rehospitalizations (POR = 1.66, 95 % CI, 1.47–1.88) after birth than infants who did not have a NICU visit (Table 2).

**Table 2:** Characteristics and outcomes, by NICU visit status.

	No./total (%)				
	Infants who had a NICU visit (n = 3287)	Infants who did not have a NICU visit (n = 28820)	Absolute difference (95 % CI)	Prevalence ratio	Prevalence odds ratio (95 % CI)
**General characteristics**					
Birth during pandemic	1675/3287 (51 %)	14858/28820 (51.6 %)	−0.60 (−2.40 to 1.21)	0.99	0.98 (0.91–1.05)
Cesarean delivery	1269/3287 (38.6 %)	8304/28820 (28.8 %)	9.79 (8.05 to 11.54)	**1.34**	**1.55 (1.44–1.67)**
Multiple delivery	337/3287 (10.3 %)	520/28820 (1.8 %)	8.45 (7.40 to 9.50)	**5.68**	**6.22 (5.39–7.17)**
**Pregnancy and labor complications**					
GDM	412/3287 (12.5 %)	3314/28820 (11.5 %)	1.04 (−0.16 to 2.23)	1.09	1.10 (0.99–1.23)
HDP	660/3287 (20.1 %)	2554/28820 (8.9 %)	11.22 (9.81 to 12.63)	**2.27**	**2.58 (2.35–2.84)**
IUGR	563/3287 (17.1 %)	2611/28820 (9.1 %)	8.07 (6.74 to 9.40)	**1.89**	**2.07 (1.88–2.29)**
PROM	792/3287 (24.1 %)	5672/28820 (19.7 %)	4.41 (2.88 to 5.95)	**1.22**	**1.30 (1.19–1.41)**
**Infant outcomes**					
GA <37 weeks	1397/3287 (42.5 %)	867/28820 (3.0 %)	39.49 (37.79 to 41.19)	**14.13**	**23.83 (21.63–26.25)**
Low birth weight	1095/3287 (33.3 %)	798/28820 (2.8 %)	30.54 (28.92 to 32.17)	**12.03**	**17.54 (15.86–19.41)**
Respiratory distress	1254/3287 (38.2 %)	180/28820 (0.6 %)	37.53 (35.86 to 39.19)	**61.08**	**98.14 (83.42–115.47)**
Meconium aspiration	69/3287 (2.1 %)	3/28820 (0.01 %)	2.09 (1.60 to 2.58)	**201.66**	**205.96 (64.79–654.73)**
Jaundice	656/3287 (20.0 %)	1039/28820 (3.6 %)	16.35 (14.97 to 17.74)	**5.54**	**6.67 (6.00–7.41)**
Pneumonia	7/3287 (0.2 %)	2/28820 (0.007 %)	0.21 (0.05 to 0.36)	**30.69**	**30.75 (6.39–148.09)**
Hypoglycemia	555/3287 (16.9 %)	450/28820 (1.6 %)	15.32 (14.03 to 16.61)	**10.81**	**12.81 (11.24–14.59)**
Hypothermia	43/3287 (1.3 %)	56/28820 (0.2 %)	1.11 (0.72 to 1.51)	**6.73**	**6.81 (4.57–10.15)**
HIE	63/3287 (1.9 %)	2/28820 (0.007 %)	1.91 (1.44 to 2.38)	**276.19**	**281.57 (68.86–1151.24)**
Sepsis	38/3287 (1.2 %)	9/28820 (0.03 %)	1.12 (0.76 to 1.49)	**37.02**	**37.44 (18.09–77.50)**
Necrotizing enterocolitis	9/3287 (0.3 %)	5/28820 (0.02 %)	0.26 (0.08 to 0.44)	**15.78**	**15.82 (5.30–47.24)**
**Health care seeking and utilization**					
Visit after discharge home within 90 days	745/3287 (22.7 %)	5851/28820 (20.3 %)	2.36 (0.86 to 3.87)	**1.12**	**1.15 (1.06–1.25)**
Rehospitalization after discharge	337/3287 (10.3 %)	1854/28820 (6.4 %)	3.82 (2.74 to 4.89)	**1.59**	**1.66 (1.47–1.88)**

NOTE: GA = gestational age; GDM = gestational diabetes; HDP = hypertensive disorder of pregnancy; HIE = hypoxic ischemia encephalopathy; IUGR = intrauterine growth restriction; PROM = premature rupture of membranes.

Infants admitted into the NICU had less hospital revisits within 90 days during the pandemic than newborns admitted to the NICU before the pandemic (19.7 % versus 25.5 %; POR = 0.77, 95 % CI, 0.61–0.85) whereas the prevalence of rehospitalization remained stable (9.8 % versus 10.7 %; POR = 0.91, 95 % CI, 0.72–1.14; Table 3). There was an increase in the prevalence of newborns with respiratory distress (41.2 % versus 35.2 %; POR = 1.29, 95 % CI, 1.12–1.48), meconium aspiration (2.8 % versus 1.4 %; POR = 1.98, 95 % CI, 1.20–3.26), and hypothermia (1.9 % versus 0.7 %; POR = 2.72, 95 % CI, 1.39–5.31) among NICU-admitted newborns during the pandemic (Table 3). The proportion of infants with hypoglycemia decreased (15.4 % versus 18.3 %; POR = 0.81, 95 % CI, 0.67–0.97; Table 3).

**Table 3:** Characteristics and outcomes for infants who had a neonatal intensive care unit visit (n = 3287).

	No./total (%)				
	Birth during pandemic	Birth before pandemic	Absolute difference (95 % CI)	Prevalence ratio	Prevalence odds ratio (95 % CI)
**General characteristics**					
Cesarean delivery	621/1612 (38.5)	648/1675 (38.7)	−0.16 (−3.49 to 3.17)	1.00	0.99 (0.86–1.14)
Multiple delivery	164/1612 (10.2)	173/1675 (10.3)	−0.15 (−2.23 to 1.92)	0.99	0.98 (0.78–1.23)
**Pregnancy and labor complications**					
GDM	205/1612 (12.7)	207/1675 (12.4)	0.36 (−1.91 to 2.62)	1.03	1.03 (0.84–1.27)
HDP	330/1612 (20.5)	330/1675 (19.7)	0.77 (−1.97 to 3.51)	1.04	1.05 (0.88–1.24)
IUGR	262/1612 (16.3)	301/1675 (18.0)	−1.72 (−4.29 to 0.86)	0.90	0.89 (0.74–1.06)
PROM	397/1612 (24.6)	395/1675 (23.6)	1.05 (−1.88 to 3.97)	1.04	1.06 (0.90–1.24)
**Infant outcomes**					
GA <37 weeks	696/1612 (43.2)	701/1675 (41.9)	1.33 (−2.06 to 4.71)	1.03	1.06 (0.92–1.21)
Low birth weight	513/1612 (31.8)	581/1675 (34.7)	−2.86 (−6.08 to 0.36)	0.92	0.88 (0.76–1.02)
Respiratory distress	664/1612 (41.2)	590/1675 (35.2)	5.97 (2.65 to 9.28)	**1.17**	**1.29 (1.12–1.48)**
Meconium aspiration	45/1612 (2.8)	24/1675 (1.4)	1.36 (0.37 to 2.34)	**1.95**	**1.98 (1.20–3.26)**
Hyperbilirubinemia	319/1612 (19.8)	337/1675 (20.1)	−0.33 (−3.06 to 2.40)	0.98	0.98 (0.83–1.16)
Pneumonia	1/1612 (0.06)	6/1675 (0.36)	−0.30 (−0.61 to 0.01)	0.17	0.17 (0.02–1.44)
Hypoglycemia	248/1612 (15.4)	307/1675 (18.3)	−2.94 (−5.50 to −0.39)	**0.84**	**0.81 (0.67–0.97)**
Hypothermia	31/1612 (1.9)	12/1675 (0.7)	1.21 (0.42 to 1.99)	**2.68**	**2.72 (1.39–5.31)**
HIE	38/1612 (2.4)	25/1675 (1.5)	0.86 (−0.08 to 1.81)	1.58	1.59 (0.96–2.65)
Sepsis	20/1612 (1.2)	18/1675 (1.1)	0.17 (−0.57 to 0.90)	1.15	1.16 (0.61–2.19)
Necrotizing enterocolitis	4/1612 (0.2)	5/1675 (0.3)	−0.05 (−0.41 to 0.31)	0.83	0.83 (0.22–3.10)
**Health care seeking and utilization**					
Visit after discharge home within 90 days	318/1612 (19.7)	427/1675 (25.5)	−5.77 (−8.62 to −2.91)	**0.77**	**0.72 (0.61–0.85)**
Rehospitalization after discharge	158/1612 (9.8)	179/1675 (10.7)	−0.89 (−2.96 to 1.19)	0.92	0.91 (0.72–1.14)

NOTE: GA = gestational age; GDM = gestational diabetes; HDP = hypertensive disorder of pregnancy; HIE = hypoxic ischemia encephalopathy; IUGR = intrauterine growth restriction; PROM = premature rupture of membranes.

### 3.3 Predictors of hospital revisit and rehospitalization

**Fig. 1:**
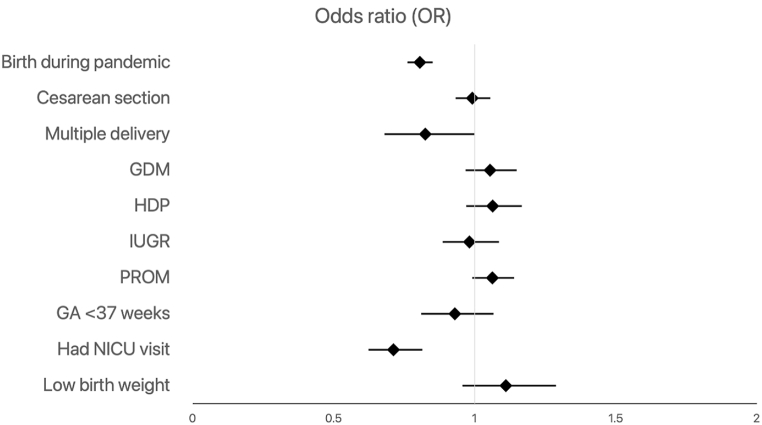
Predictors of hospital revisit among all infants after discharge home within 90 days (GA = gestational age; GDM = gestational diabetes; HDP = hypertensive disorder of pregnancy; IUGR = intrauterine growth restriction; PROM = premature rupture of membranes; NICU = neonatal intensive care unit).

**Fig. 2:**
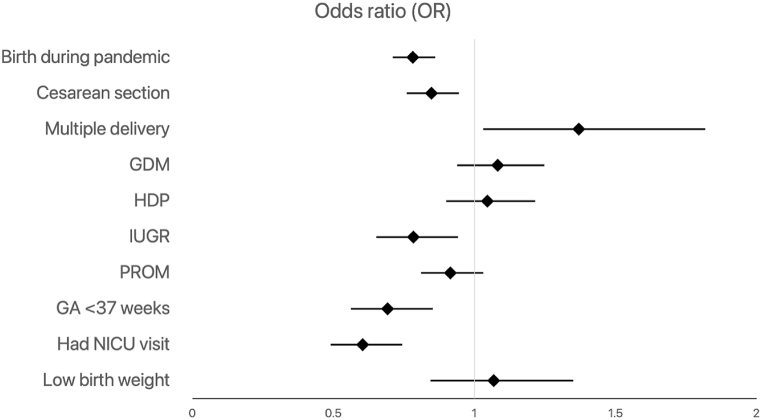
Predictors of rehospitalization among all infants after discharge home within 90 days (GA = gestational age; GDM = gestational diabetes; HDP = hypertensive disorder of pregnancy; IUGR = intrauterine growth restriction; PROM = premature rupture of membranes; NICU = neonatal intensive care unit).

Among all infants, birth during the pandemic (OR = 0.81; 95 % CI, 0.76–0.85) and NICU visit (OR = 0.71; 95 % CI, 0.62–0.82) resulted in lower odds of hospital revisit within 90 days (Fig. 1, Supplementary Table A). Birth during the pandemic (OR = 0.78; 95 % CI, 0.71–0.86), NICU visit (OR = 0.60; 95 % CI, 0.49–0.74), and gestational age less than 37 weeks (OR = 0.69; 95 % CI, 0.56–0.85) resulted in lower odds of rehospitalization (Fig. 2, Supplementary Table A).

**Fig. 3:**
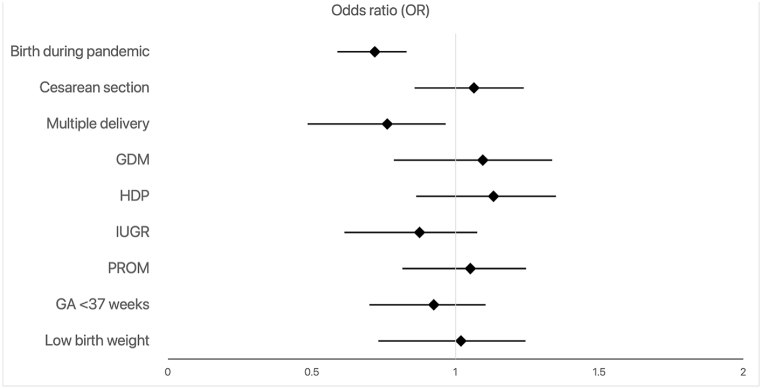
Predictors of hospital revisit among NICU-admitted infants after discharge home within 90 days (GA = gestational age; GDM = gestational diabetes; HDP = hypertensive disorder of pregnancy; IUGR = intrauterine growth restriction; PROM = premature rupture of membranes; NICU = neonatal intensive care unit).

**Fig. 4:**
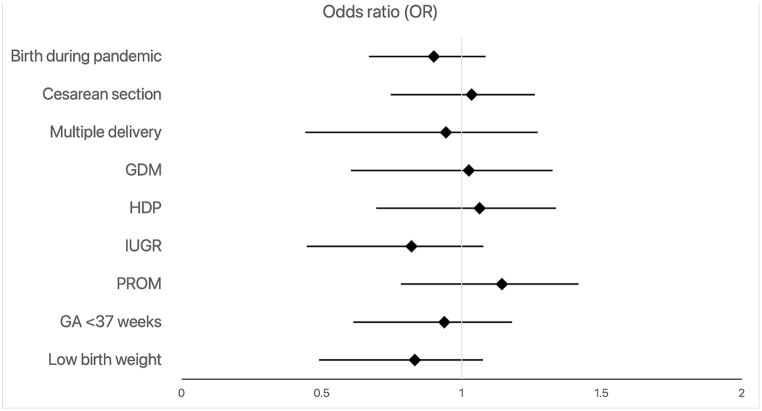
Predictors of rehospitalization among NICU-admitted infants after discharge home within 90 days (GA = gestational age; GDM = gestational diabetes; HDP = hypertensive disorder of pregnancy; IUGR = intrauterine growth restriction; PROM = premature rupture of membranes; NICU = neonatal intensive care unit).

For infants who had a NICU visit, birth during the pandemic resulted in lower odds of hospital revisit within 90 days following discharge (OR = 0.90; 95 % CI, 0.72–1.13; Fig. 3 and Supplementary Table B) while the odds of rehospitalization were similar for both time periods Fig. 4 and Supplementary Table B).

### 3.4 Mother and newborn outcomes by COVID-19 status

In the sample, 11 (0.03 %) infants had confirmed COVID-19 at birth and 37 (0.12 %) infants were born to persons who had confirmed COVID-19 at time of labor (large confidence intervals are attributable to the small sample size of infected children or infected mothers; Table 4). No infants had confirmed COVID-19 while also having a mother who had confirmed COVID-19. Infants who had confirmed COVID-19 at birth were more likely to have a hospital revisit within 90 days (90.9 % versus 20.5 %; PR = 4.43; POR = 38.73, 95 % CI, 4.96–302.64) and further be re-hospitalized after discharge (90.9 % versus 6.8 %; PR = 13.38; POR = 137.16, 95 % CI, 17.55–1072.01; Table 4). Characteristics of pregnancy and labour as well as infant outcomes were similar between infants with and without confirmed COVID-19 at birth (Table 4). For infants born to persons with confirmed COVID-19 at time of labor, rates of hospital revisits and rehospitalization were similar (Table 5). For this group, a greater prevalence of preterm birth (18.9 % versus 7.0 %; PR = 2.69; POR = 3.08, 95 % CI, 1.35–7.02) was observed compared to infants born to persons without confirmed COVID-19 (Table 5).

**Table 4:** Characteristics and outcomes, by COVID-19 status in infants.

	No./total (%)				
	Infants with confirmed COVID-19 at birth (n = 11)	Infants without confirmed COVID-19 at birth (n = 32096)	Absolute difference (95 % CI)	Prevalence ratio	Prevalence odds ratio (95 % CI)
**General characteristics**					
Cesarean delivery	6/11 (54.5)	9567/32096 (29.8)	24.74 (−4.69 to 54.17)	1.83	2.83 (0.86–9.26)
Multiple delivery	0/11 (0)	857/32096 (2.7)	−2.67 (−2.85 to −2.49)	–	–
**Pregnancy and labor complications**					
GDM	3/11 (27.3)	3723/32096 (11.6)	15.67 (−10.65 to 41.99)	2.35	2.86 (0.76–10.78)
HDP	0/11 (0)	3214/32096 (10)	−10.01 (−10.34 to −9.69)	–	–
IUGR	1/11 (9.1)	3173/32096 (9.9)	−0.80 (−17.79 to 16.20)	0.92	0.91 (0.12–7.12)
PROM	2/11 (18.2)	6462/32096 (20.1)	−1.95 (−24.75 to 20.85)	0.90	0.88 (0.19–4.08)
**Infant outcomes**					
NICU visit	0/11 (0)	3287/32096 (10.2)	−10.24 (−10.57 to −9.91)	–	–
GA <37 weeks	1/11 (9.1)	2263/32096 (7.1)	2.04 (−14.95 to 19.03)	1.29	1.32 (0.17–10.30)
Low birthweight	0/11 (0)	1893/32096 (5.9)	−5.90 (−6.16 to −5.64)	–	–
Respiratory distress	1/11 (9.1)	1433/32096 (4.5)	4.63 (−12.36 to 21.62)	2.04	2.14 (0.27–16.73)
Meconium aspiration	0/11 (0)	72/32096 (0.2)	−0.22 (−0.28 to −0.17)	–	–
Jaundice	0/11 (0)	1695/32096 (5.3)	−5.28 (−5.53 to −5.04)	–	–
Pneumonia	0/11 (0)	9/32096 (0.03)	−0.03 (−0.05 to −0.01)	–	–
Hypoglycemia	0/11 (0)	1005/32096 (3.1)	−3.13 (−3.32 to −2.94)	–	–
Hypothermia	0/11 (0)	99/32096 (0.3)	−0.31 (−0.37 to −0.25)	–	–
HIE	0/11 (0)	65/32096 (0.2)	−0.20 (−0.25 to −0.15)	–	–
Sepsis	0/11 (0)	47/32096 (0.15)	−0.15 (−0.19 to −0.10)	–	–
Necrotizing enterocolitis	0/11 (0)	14/32096 (0.04)	−0.04 (−0.07 to −0.02)	–	–
**Health care seeking and utilization**					
Visit after discharge home within 90 days	10/11 (90.9)	6586/32096 (20.5)	70.39 (53.39 to 87.38)	**4.43**	**38.73 (4.96–302.64)**
Rehospitalization after discharge	10/11 (90.9)	2181/32096 (6.8)	84.11 (67.12 to 101.11)	**13.38**	**137.16 (17.55–1072.01)**

NOTE: GA = gestational age; GDM = gestational diabetes; HDP = hypertensive disorder of pregnancy; HIE = hypoxic ischemia encephalopathy; IUGR = intrauterine growth restriction; PROM = premature rupture of membranes.

**Table 5:** Characteristics and outcomes, by COVID-19 status in pregnant persons.

	No./total (%)				
	Infants born to persons with confirmed COVID-19 at time of labor (n = 37)	Infants born to persons without confirmed COVID-19 at time of labor (n = 32070)	Absolute difference (95 % CI)	Prevalence ratio	Prevalence odds ratio (95 % CI)
**General characteristics**					
Cesarean delivery	13/37 (35.1)	9560/32070 (29.8)	5.33 (−10.07 to 20.72)	1.18	1.28 (0.65–2.51)
Multiple delivery	2/37 (5.4)	855/32070 (2.7)	2.74 (−4.55 to 10.03)	2.03	2.09 (0.50–8.69)
**Pregnancy and labor complications**					
GDM	7/37 (18.9)	3719/32070 (11.6)	7.32 (−5.30 to 19.95)	1.63	1.78 (0.78–4.05)
HDP	6/37 (16.2)	3208/32070 (10)	6.21 (−5.67 to 18.09)	1.62	1.74 (0.73–4.18)
IUGR	3/37 (8.1)	3171/32070 (9.9)	−1.78 (−10.58 to 7.02)	0.82	0.80 (0.25–2.62)
PROM	8/37 (21.6)	6456/32070 (20.1)	1.49 (−11.78 to 14.76)	1.07	1.09 (0.50–2.40)
**Infant outcomes**					
NICU visit	5/37 (13.5)	3282/32070 (10.2)	3.28 (−7.74 to 14.30)	1.32	1.37 (0.53–3.52)
GA <37 weeks	7/37 (18.9)	2257/32070 (7.0)	11.88 (−0.74 to 24.50)	**2.69**	**3.08 (1.35–7.02)**
Low birth weight	3/37 (8.1)	1890/32070 (5.9)	2.21 (−6.58 to 11.01)	1.38	1.41 (0.43–4.59)
Respiratory distress	3/37 (8.1)	1431/32070 (4.5)	3.65 (−5.15 to 12.44)	1.82	1.89 (0.58–6.16)
Meconium aspiration	0/37 (0)	72/32070 (0.2)	−0.22 (−0.28 to −0.17)	–	–
Jaundice	0/37 (0)	1695/32070 (5.3)	−5.29 (−5.53 to −5.04)	–	–
Pneumonia	0/37 (0)	9/32070 (0.03)	−0.03 (−0.05 to −0.01)	–	–
Hypoglycemia	1/37 (2.7)	1004/32070 (3.1)	−0.43 (−5.66 to 4.80)	0.86	0.86 (0.12–6.28)
Hypothermia	1/37 (2.7)	98/32070 (0.3)	2.40 (−2.83 to 7.62)	8.84	9.06 (1.23–66.76)
HIE	0/37 (0)	65/32070 (0.2)	−0.20 (−0.25 to −0.15)	–	–
Sepsis	0/37 (0)	47/32070 (0.15)	−0.15 (−0.19 to −0.10)	–	–
Necrotizing enterocolitis	0/37 (0)	41/32070 (0.13)	−0.13 (−0.17 to −0.09)	–	–
**Health care seeking and utilization**					
Visit after discharge home within 90 days	7/37 (18.9)	6589/32070 (20.5)	−1.63 (−14.25 to 11.00)	0.92	0.90 (0.40–2.06)
Rehospitalization after discharge	3/37 (8.1)	2188/32070 (6.8)	1.29 (−7.51 to 10.09)	1.19	1.21 (0.37–3.93)

NOTE: GA = gestational age; GDM = gestational diabetes; HDP = hypertensive disorder of pregnancy; HIE = hypoxic ischemia encephalopathy; IUGR = intrauterine growth restriction; PROM = premature rupture of membranes.

## 4 Discussion

This appears to be the first province-wide Albertan study that reports health conditions of mothers and their newborns one year prior to and into the pandemic, while also considering predictors of hospital revisits, hospital rehospitalizations, and NICU admissions for neonates born during the COVID-19 pandemic. It also examined health outcomes of NICU-admitted newborns and the impact that COVID-19 infection had on maternal and newborn outcomes. Most of the findings are contextualized on pandemic-related stressors (rather than COVID-19 infection) and contrast those from low-to-middle income countries. In general, characteristics were comparable for those born prior to and during the pandemic, suggesting that Albertan hospitals adapted well to the pandemic. However, some differences were observed in relation to hospital visits and rehospitalization after discharge (i.e., while visits after discharge decreased for infants who had a NICU visit, rehospitalization remained stable). NICU newborns experienced higher prevalence of all adverse health outcomes compared to those not admitted to the NICU. Further, NICU-admitted pandemic newborns exhibited more of some respiratory health conditions compared to NICU-admitted newborns before the pandemic. Our findings show some similarity to findings from other Canadian studies; however other Canadian studies often generalized children's health conditions as a broader category rather than describing specific conditions or described fewer health conditions than described in our study [[26], [27], [28]]. Therefore, findings from this study help to bridge these gaps and illuminate novel outcomes in Canada.

Most characteristics of mothers and infants from all births remained the same with the onset of the pandemic in our study, paralleling findings deriving from other high-income countries [29]. However, some differences were observed. For example, infants born during the pandemic had greater birthweights compared to their non-pandemic counterparts, contrasting the decrease that was expected to occur with the numerous novel pandemic-related stressors [30]; greater birthweight was also observed by Li et al. in their study of COVID-19 impacts on child birthweight in China [31]. Further, hypertensive disorders during pregnancy (reflecting a broader composition of high blood pressure conditions) slightly increased during the one-year pandemic period. This finding was expected given the general decrease in physical activity stemming from pandemic-related restrictions and changes in lifestyle habits (e.g., less physical activity, changes in nutritional intake) [32]. Lower physical activity and poorer nutritional intake could also explain the greater observed birthweight of newborns born during the pandemic [32]. Nevertheless, to better understand these outcomes, additional data pertaining to body mass index and nutritional factors should be collected and examined, revealing an area for future research.

Another positive outcome that was observed was that there was no significant change in the number of NICU admissions prior to and during the COVID-19 pandemic, again opposite to that which was hypothesized, but paralleling findings deriving from other high-income countries [27]. In their meta-analysis, Chmielewska et al. reported a pooled OR of 0.91 [0.80 to 1.03] for the odds of NICU admission with the onset of the pandemic when combining data deriving from six high-income country studies [29]. The Canadian government provided some relief to families by providing financial support and other resources (particularly in the first year of the pandemic) which may have helped to buffer the expected negative impacts of stress on antenatal development in this region [33]. Data in this study did also reveal that the number of preterm birth and low birthweight infants remained comparable before and during the pandemic in this region, which may further support why NICU admissions remained comparable between the two time periods.

As expected, among all births, preterm birth and low birthweight infants were strongly predictive of NICU admission in our study. This aligns with existing literature which affirms that preterm birth and low birthweight (often comorbid in nature) result in developmental challenges that undermine health and require intensive care [3]. Cesarean section also predicted NICU admissions, a procedure that is known to increase the risk of newborn health complications (e.g., transient tachypnoea of newborn, respiratory distress syndrome) that require NICU monitoring [3,34]. When examining health conditions of mothers, pre-existing diabetes and hypertensive disorders during pregnancy predicted NICU admission. These maternal conditions are known to be indirectly comorbid with respiratory syndromes in children [3], among others, which might explain why access to NICU was required for these newborns. Identification of these predictors can guide healthcare professionals in hospital settings to address impacts during pregnancy and reduce the likelihood of NICU admission or inordinate hospitalization utilization.

Newborns admitted to the NICU had a statistically higher odds of all adverse health outcomes examined in this study compared to newborns with no NICU admission. These findings, though alarming, align with the purpose and environment of the NICU, which aims to immediately support newborns who require intensive care for various health conditions [35]. However, when comparing outcomes between newborns admitted into the NICU prior to and during the pandemic did, the prevalence of negative impacts was even higher for certain conditions among those born during the pandemic. As observed among all births, NICU-admitted infants born during the pandemic had lower birthweight; this parallels other literature as NICU-admitted newborns are often characterized by lower than normal birthweights [36,37]. On the other hand, NICU-admitted infants born during the pandemic had more cases of transient tachypnoea of newborn and meconium aspiration syndrome. At first glance, these outcomes may appear to arise from the effects of COVID-19 on respiratory health, however, is more likely attributable to effects from prenatal stress (which is known to undermine respiratory development) given the low prevalence of COVID-19 infection in our study [38]. Although these three factors were greater among NICU-admitted infants born during the pandemic, the clinical significance appears small, suggesting that the pandemic had little effects on newborns admitted to the NICU compared to those admitted to the NICU prior to the pandemic.

Pandemic births also predicted lower odds of hospital revisits after discharge from NICU in this study. The aforementioned reasons regarding why mothers did not revisit hospitals during the pandemic may help to explain this outcome (i.e., fear of COVID-19 infection, lack of social support) [39]. Further, necrotizing enterocolitis served as a risk factor (broad confidence interval indicates decreased precision around the estimate) for hospital revisits in this study whereas jaundice/hyperbilirubinemia predicted both hospital revisits and rehospitalizations following NICU discharge. The life-threatening characteristic of this condition, in addition to its more evident physical manifestation [40], is likely why mothers returned with their newborn to the hospital. Unexpectedly, lower birthweight predicted decreased odds of hospital readmission following NICU discharge. This may imply that infants born at lower weights than average received the care required within the NICU setting prior to discharge. While all maternal and newborn characteristics interact to influence NICU outcomes, these findings emphasize factors that may be most influential.

In this study, the number of hospital revisits and rehospitalizations appeared to decrease during the pandemic compared to infants born prior, supporting the study's hypothesis about hospital utilization. These findings are similar to those from others, such as To et al.’s population-based cohort study conducted in Ontario, Canada [41]. Lower hospital utilization may suggest that at a broader level, Albertan hospitals adapted well to the COVID-19 pandemic. Conversely, it may also indicate that mothers and families were more cautious when accessing hospitals following discharge due to fears and anxieties of contracting COVID-19, thus carefully choosing to access tertiary healthcare settings only when deemed necessary [39]. Further, due to novel policies such as restricted visitor access and decreased social support within hospital settings, mothers may have preferred to remain at home and self-manage their health concerns or access other services such as primary care offices instead [39]. From a population health perspective, it must be noted that hospital use behaviours likely intersected with individuals' identities, as literature reinforces that sexual, gender, ethnic, and racial minority groups express hesitancy in accessing healthcare due to experiences of oppression, discrimination, racism, stigma, and lack of inclusivity which lead to health inequities [42]. Stratifying patterns of healthcare use by social determinants of health may demonstrate underlying social forces that impact healthcare use concomitant to those of COVID-19, which would help to guide future public health measures.

Adverse birth outcomes predicted higher hospital use in this study, which is similar to the results from other studies [43]. Children born preterm are at greater risk of developmental delay, and in turn, lifelong health consequences [3]. This implies an increased likelihood of experiencing poorer health in the newborn period which may increase hospital utilization [3]. In addition to co-morbidity with other health conditions (e.g., stroke), hypertensive disorders during pregnancy can result in severe health complications after birth, which warrant immediate and professional support [44]. To reduce the need for hospital revisits or rehospitalizations, obstetricians and nurses should create carefully planned support packages, ensure follow-up is performed, and link women who had hypertensive disorders during pregnancy or preterm infants to primary care services.

Interestingly, findings from this study revealed no differences in health outcomes for newborns born with COVID-19; however, newborns with COVID-19 did have more hospital revisits and rehospitalization after discharge and newborns born to mothers with COVID-19 were more likely to be born preterm than newborns born to mothers without COVID-19. These findings mainly oppose that which was hypothesized, however, must be interpreted with caution due to the small sample sizes available for data analysis. Allotey et al. conducted a meta-analysis of over 400 studies which included 800 fetuses/newborns testing positive for COVID-19 (with available outcome data); of those, approximately 6 % were lost due to either stillbirths, neonatal deaths, or early pregnancy loss [45]. It is important to note that these authors observed a positivity rate of only 0.1 % [0.0 %–0.3 %] in North America after pooling findings from various regions [45]. Another study demonstrated how COVID-19 infection among mothers resulted in preterm birth [46], paralleling findings from our study. Collectively, despite the small sample size in this study, findings from our study parallel those from other studies conducted in high-income countries [23]. To counteract the potential implications of preterm births from COVID-19 infection among mothers, healthcare professionals should ensure to monitor child development through screening assessments and provide mothers with tools and resources to promote their child's development [47].

In general, findings from this study, situated within a high-income country with public and integrated health care systems in context, are mostly reassuring. This can be concluded since: 1) similar prevalence of complications and outcomes before and during the pandemic was observed; 2) similar prevalence of rehospitalization before and during the pandemic for NICU-admitted infants was observed; and 3) many adverse outcomes were not observed in the COVID-19 infected subgroups (with acknowledgement of the limitations given how small these subgroups are). Chmielewska et al. revealed fewer significant differences in mother-newborn outcomes with the onset of the pandemic in high-income countries when compared to low-income countries [29]. However, findings from some low-to-moderate income countries, such as those from Ranjbar et al.’s study conducted in Iran [48], parallel those from our study, suggesting that other factors (e.g., sociopolitical approaches to the COVID-19 pandemic) should also be considered beyond only the wealth of a country when examining effects of COVID-19 on mothers and their newborns. Nevertheless, our findings, which derive from a high-income country, generally paralleled those from other high-income countries and contrasted those from low-to-moderate income countries [23,29], alluding to the protective effects of residing in a high-income country as opposed to low-to-moderate income countries during the COVID-19 pandemic and the importance of having adequate resources and services in the case of such a healthcare emergency.

### 4.1 Recommendations and policy implications

The COVID-19 pandemic blindsided every country globally, however, findings from this study reveal some resiliency in terms of maternal-newborn health and implications for policy and the promotion of public health. Firstly, the provision of financial relief to pregnant mothers is needed to increase mothers' capacity to provide quality care to their newborns and to reduce stress. This may be particularly important for single pregnant mothers as employment among women was disproportionately (and unequally) impacted by the pandemic, with higher rates of job loss [49]. This policy recommendation must be emphasized further in relation to the social determinants of health, providing a graded financial relief (rather than a lump sum to all) for mothers who care for more than one child, those with varying physical and mental abilities, and those who are marginalized to increase health equity and maximize their infants' health. Secondly, an integration of a robust post-pandemic monitoring system is needed for infants born in similar conditions to decrease the potentiality for repercussions on development and longer-term health outcomes. This primary approach to health prevention can potentially decrease the health sequelae that may occur and optimize lifelong health outcomes. For newborns who developed respiratory health conditions, this recommendation is even more significant. Lastly, there needs to be an emphasis on determining causal factors for lower hospital utilization and greater birthweights during the COVID-19 pandemic. This includes examining whether other healthcare settings were preferred such as primary care settings; doing so can reveal how pandemic restrictions may have prevented mothers’ from accessing tertiary levels of care and evaluate whether mothers were in fact accessing healthcare settings when needed.

### 4.2 Strengths and limitations

Given how rapidly COVID-19 evolved in the first year, the one-year span collected during the pandemic may not be reflective of a static effect of the pandemic on child outcomes. Nevertheless, the first year was comprised of the most changes in Canada, and therefore, this study reveals important COVID-19 implications on perinatal health. Secondly, sociodemographic variables were not collected, limiting an in-depth exploration from a population health perspective. Some comorbidities may have also been missed; however, this was beyond the scope of the paper. Most of the differences observed among health conditions are difficult to interpret as being clinically significant, but they are still important to highlight and there appears to be clinical relevance in hospital utilization patterns alongside health conditions when comparing NICU-admitted versus non-NICU-admitted newborns, epitomizing the importance of this study. Despite these limitations, this study had several strengths. Firstly, the sample size of this study is very large and concentrated in a small region of North America, enabling some level of generalizability in similar regions (e.g., similar health care system). Another strength is that this study was provincially based, allowing for what appears to be the first province wide study examining the effects of COVID-19 on Albertan mothers and their newborns. Lastly, we had access to numerous variables, allowing for our team to conduct multiple regression analyses.

## 5 Conclusions

This novel study elucidates findings using a large dataset (n > 32,000) regarding the impacts of the COVID-19 pandemic on maternal and newborn health in Alberta, Canada. This study included hospital use patterns that can be helpful in identifying potential concerns with resource management during the pandemic. Birthweights increased during the pandemic and timing of birth appeared to play an important role, whereby birth during the pandemic served as a protective factor to hospital readmission and hospital revisits. Some respiratory conditions, along with hyperbilirubinemia, appeared to be prominent predictors of hospital revisits and rehospitalizations following NICU discharge. Most of the findings are contextualized on pandemic-related stressors (rather than COVID-19 infection) and are likely more generalizable to high-income countries. Overall, decreases in hospital use behaviours and increases in newborn respiratory conditions need to be concomitantly investigated alongside sociodemographic and pandemic-related factors (such as fears of contracting COVID-19 or viral interactions with other unintended pandemic outcomes), respectively, to ensure that patterns are being interpreted accurately and are reflective of the socioeconomic, political, and medical interactions resulting from the pandemic.

## Funding source

This research did not receive any specific grant from funding agencies in the public, commercial, or not-for-profit sectors.

## Data availability statement

Data associated with this study have not been deposited into a publicly available repository, however, data will be made available on request.

## CRediT authorship contribution statement

**Stefan Kurbatfinski:** Writing – review & editing, Writing – original draft, Supervision, Project administration, Methodology, Investigation, Data curation, Conceptualization. **Aliyah Dosani:** Writing – review & editing, Supervision, Project administration, Methodology, Investigation, Funding acquisition, Data curation, Conceptualization. **Carlos Fajardo:** Writing – review & editing, Methodology, Investigation, Funding acquisition, Data curation, Conceptualization. **Alexander Cuncannon:** Writing – review & editing, Methodology, Formal analysis, Data curation. **Aliza Kassam:** Writing – review & editing. **Abhay K. Lodha:** Writing – review & editing, Resources, Methodology, Funding acquisition, Data curation, Conceptualization.

## Declaration of competing interest

The authors declare that they have no known competing financial interests or personal relationships that could have appeared to influence the work reported in this paper.
