# Supplementary material for: Impacts of COVID-19 on mothers’ and newborns’ health outcomes in regional Canada: A cross-sectional analysis
Source: Heliyon. 2024 Jul 5;10(14):e34165. doi: 10.1016/j.heliyon.2024.e34165 (PMC11292244; doi:10.1016/j.heliyon.2024.e34165)
Supplement: Multimedia component 1 [file mmc1.docx]

Supplementary Table A: Model of predictors of hospital revisit and re-hospitalization among all infants (n=32107)

|  | Visit after discharge home within 90 days (6596/32107) | Re-hospitalization after discharge (2191/32107) |
| --- | --- | --- |
|  | Odds ratio (95% CI) | Odds ratio (95% CI) |
| General characteristics | | |
| Birth during pandemic | 0.805 (0.762-0.852)* | 0.782 (0.71-0.86)* |
| Cesarean delivery | 0.992 (0.932-1.055) | 0.847 (0.76-0.945) |
| Multiple delivery | 0.824 (0.681-0.999) | 1.369 (1.031-1.818) |
| Pregnancy and labor complications | | |
| GDM | 1.054 (0.968-1.149) | 1.082 (0.938-1.248) |
| HDP | 1.064 (0.97-1.167) | 1.045 (0.9-1.215) |
| IUGR | 0.982 (0.887-1.086) | 0.783 (0.652-0.941) |
| PROM | 1.063 (0.991-1.139) | 0.914 (0.811-1.031) |
| Infant outcomes | | |
| NICU visit | 0.712 (0.623-0.815)* | 0.604 (0.49-0.743)* |
| GA <37 weeks | 0.93 (0.811-1.066) | 0.692 (0.562-0.852)* |
| Low birth weight | 1.11 (0.957-1.288) | 1.068 (0.844-1.35) |
| Respiratory distress | 1.074 (0.912-1.266) | 1.093 (0.854-1.398) |
| Meconium aspiration | 0.977 (0.523-1.824) | 0.564 (0.184-1.728) |
| Jaundice | 5.994 (5.38-6.679)* | 24.254 (21.356-27.545)* |
| Pneumonia | 2.975 (0.705-12.542) | 0.802 (0.112-5.737) |
| Hypoglycemia | 1.15 (0.978-1.353) | 0.964 (0.744-1.251) |
| Hypothermia | 4.572 (3.011-6.943)* | 14.439 (9.14-22.81)* |
| HIE | 1.165 (0.627-2.163) | 1.369 (0.544-3.448) |
| Sepsis | 2.033 (1.061-3.897) | 7.005 (3.277-14.971)* |
| Necrotizing enterocolitis | 14.728 (3.949-54.924)* | 21.835 (6.588-72.732)* |

*p<0.001

GA, gestational age; GDM, gestational diabetes mellitus; HDP, hypertensive disorder of pregnancy; HIE, hypoxic ischemic encephalopathy; IUGR, intrauterine growth restriction; NICU, neonatal intensive care unit; PROM, premature rupture of membranes

Supplementary Table B: Model of predictors of hospital revisit and re-hospitalization among infants admitted to NICU (n=3287)

|  | Visit after discharge home within 90 days (745/3287) | Re-hospitalization after discharge (337/3287) |
| --- | --- | --- |
|  | Odds ratio (95% CI) | Odds ratio (95% CI) |
| General characteristics | | |
| Birth during pandemic | 0.719 (0.609-0.849)* | 0.901 (0.717-1.133) |
| Cesarean delivery | 1.064 (0.891-1.27) | 1.036 (0.81-1.325) |
| Multiple delivery | 0.762 (0.559-1.039) | 0.944 (0.616-1.446) |
| Pregnancy and labor complications | | |
| GDM | 1.095 (0.853-1.404) | 1.026 (0.727-1.447) |
| HDP | 1.132 (0.915-1.401) | 1.063 (0.79-1.433) |
| IUGR | 0.874 (0.673-1.135) | 0.821 (0.564-1.195) |
| PROM | 1.051 (0.858-1.288) | 1.144 (0.87-1.504) |
| Infant outcomes | | |
| GA <37 weeks | 0.92 (0.744-1.148) | 0.938 (0.697-1.263) |
| Low birth weight | 1.019 (0.795-1.306) | 0.833 (0.59-1.175) |
| Respiratory distress | 0.968 (0.809-1.157) | 0.866 (0.676-1.11) |
| Meconium aspiration | 0.909 (0.497-1.661) | 0.519 (0.185-1.453) |
| Jaundice | 1.217 (0.991-1.494) | 1.567 (1.199-2.048) |
| Pneumonia | 2.083 (0.444-9.779) | 0.742 (0.081-6.799) |
| Hypoglycemia | 1.199 (0.962-1.494) | 1.00 (0.732-1.366) |
| Hypothermia | 1.528 (0.783-2.982) | 2.11 (0.954-4.667) |
| HIE | 1.051 (0.579-1.91) | 1.033 (0.46-2.322) |
| Sepsis | 1.233 (0.587-2.588) | 2.647 (1.204-5.822) |
| Necrotizing enterocolitis | 7.127 (1.74-29.199) | 3.897 (0.94-16.153) |

*p<0.001

GA, gestational age; GDM, gestational diabetes mellitus; HDP, hypertensive disorder of pregnancy; HIE, hypoxic ischemic encephalopathy; IUGR, intrauterine growth restriction; NICU, neonatal intensive care unit; PROM, premature rupture of membranes
